# The Effectiveness and Cost-Effectiveness of Screening for and Vaccination Against Hepatitis B Virus among Migrants in the EU/EEA: A Systematic Review

**DOI:** 10.3390/ijerph15091898

**Published:** 2018-09-01

**Authors:** Daniel T Myran, Rachael Morton, Beverly-Ann Biggs, Irene Veldhuijzen, Francesco Castelli, Anh Tran, Lukas P Staub, Eric Agbata, Prinon Rahman, Manish Pareek, Teymur Noori, Kevin Pottie

**Affiliations:** 1 University of Ottawa School of Epidemiology and Public Health, Ottawa, ON K1G 5Z3, Canada; dmyra088@uottawa.ca; 2 NHMRC Clinical Trials Centre, The University of Sydney, Sydney 2006, Australia; Rachael.morton@ctc.usyd.edu.au (R.M.); ahn.tran@ctc.usyd.edu.ar (A.T.); lukas.staub@ctc.usyd.edu.au (L.P.S.); 3 Department of Medicine at the Doherty Institute, University of Melbourne, and Victorian Infectious Diseases Service, Royal Melbourne Hospital, Melbourne 3000, Australia; babiggs@unimelb.edu.au; 4 Centre for Infectious Disease Control, National Institute for Public Health and the Environment (RIVM), Bilthoven 3720, The Netherlands; irene.veldhuijzen@rivm.nl; 5 University Department of Infectious and Tropical Diseases University of Brescia and ASST Spedali Civili, Brescia 25123, Italy; francesco.castelli@unibs.it; 6 Department of Paediatrics, Obstetrics, Gynaecology and Preventive Medicine, Universität Autònoma de Barcelona, Barcelona 08193, Spain; ricagbata@yahoo.com; 7 C.T. Lamont Primary Health Care Research Centre, Bruyère Research Institute, Ottawa, ON K1R 7G5, Canada; prinon.rahman@dal.ca; 8 Department of Infection, Immunity and Inflammation, University of Leicester, Leicester LE1 7RH, UK; manish.pareek@leicester.ac.uk; 9 European Centre for Disease Prevention and Control, Stockholm 169 73, Sweden; Teymur.Noori@ecdc.europa.eu; 10 Bruyere Research Institute, School of Epidemiology and Public Health, University of Ottawa, Ottawa, ON K1R 7G5, Canada

**Keywords:** HBV, CHB, screening, vaccination, refugees, migrants

## Abstract

Migrants from hepatitis B virus (HBV) endemic countries to the European Union/European Economic Area (EU/EEA) comprise 5.1% of the total EU/EEA population but account for 25% of total chronic Hepatitis B (CHB) infection. Migrants from high HBV prevalence regions are at the highest risk for CHB morbidity. These migrants are at risk of late detection of CHB complications; mortality and onwards transmission. The aim of this systematic review is to evaluate the effectiveness and cost-effectiveness of CHB screening and vaccination programs among migrants to the EU/EEA. We found no RCTs or direct evidence evaluating the effectiveness of CHB screening on morbidity and mortality of migrants. We therefore used a systematic evidence chain approach to identify studies relevant to screening and prevention programs; testing, treatment, and vaccination. We identified four systematic reviews and five additional studies and guidelines that reported on screening and vaccination effectiveness. Studies reported that vaccination programs were highly effective at reducing the prevalence of CHB in children (RR 0.07 95% CI 0.04 to 0.13) following vaccination. Two meta-analyses of therapy for chronic HBV infection found improvement in clinical outcomes and intermediate markers of disease. We identified nine studies examining the cost-effectiveness of screening for CHB: a strategy of screening and treating CHB compared to no screening. The median acceptance of HB screening was 87.4% (range 32.3–100%). Multiple studies highlighted barriers to and the absence of effective strategies to ensure linkage of treatment and care for migrants with CHB. In conclusion, screening of high-risk children and adults and vaccination of susceptible children, combined with treatment of CHB infection in migrants, are promising and cost-effective interventions, but linkage to treatment requires more attention.

## 1. Introduction

Hepatitis B Virus (HBV) infection can cause acute and chronic hepatitis and lead to life threatening liver complications including cirrhosis and hepatocellular carcinoma (HCC) [1]. Infection with chronic hepatitis B (CHB) is typically asymptomatic until health complications develop. However, individuals with CHB, including asymptomatic cases, can spread the virus through sexual contact, blood-blood contact, and mother to child transmission [1]. Six percent of the world’s population, approximately 248 million people, are infected with CHB [2]. The global distribution of HBV is highly variable and regions are characterized by the prevalence of Hepatitis B surface Antigen (HBsAg) as low (<2%), or endemic (≥2%) [2]. In the European Union/European Economic Area (EU/EEA) an estimated 4–7.5 million people are chronically infected with HBV, with an overall prevalence of 1.12% [3,4]. The majority of countries in the EU/EEA have a low prevalence of HBV infection, although in 2013 five countries had a prevalence >2% [3]. However, CHB infection remains a leading cause of chronic liver disease and liver cancer in the EU/EEA, and results in significant economic burden and lost productivity [5,6].

Migrants to the EU/EEA originating from endemic countries suffer a disproportionate burden of CHB, constituting 5.1% of the population but comprising an estimated 25% (range 14–47%) of total CHB cases [3]. Antenatal screening programs in the EU/EEA report that migrant women account for 1.0% to 15.4% of diagnoses of CHB, on average, six times higher than in the general female population [7]. The risk of CHB infection varies amongst different migrant groups. A systematic review of migrants throughout the world found higher prevalence of CHB in refugees, asylum seekers and migrants originating from HBV endemic countries [8].

As the majority of cases of CHB are asymptomatic, screening is important to identify individuals at risk of progression to complications and who are at risk of transmitting the virus. In addition, screening can identify individuals who are susceptible to infection and would benefit from vaccination. An effective HB vaccine has been available for nearly four decades and has reduced the incidence of new infections in the EU/EEA and globally [1,6,9,10]. Universal childhood vaccination against HB is recommended in 27/31 EU/EEA countries, and all countries recommend vaccination for children in high-risk groups, including migrants [11]. However, identification of migrants susceptible to HB infection and delivery of vaccination can be complicated. Internationally, several strategies for vaccination programs exist including universal childhood vaccination, targeted vaccination of individuals found to be susceptible to HB, and ring vaccination strategies that prevent the spread of disease by vaccinating close contacts of known HBV cases [1,12]. Individuals infected during childhood are at higher risk of developing CHB infection and complications than individually infected later in life [1,13]. Consequently, prevention of childhood HB infection in migrant communities is a public health priority.

The World Health Organization (WHO) has set the goal of eliminating CHB as a major public health threat by 2030 with targets to reduce the incidence of chronic infection by 90% and mortality by 65% [14]. National programs should include migrants from endemic countries as an important component of this global strategy. Screening programs for CHB vary by country; at present the majority of EU/EEA countries do not recommend screening for CHB in migrants [15]. Detecting CHB infections in migrants from endemic countries and subsequent management including treatment and behavioral change counselling is likely to decrease the burden of disease in the EU/EEA. In this systematic review, we aim to evaluate the effectiveness, costs (resource requirements), and cost-effectiveness of CHB screening and vaccination programs for migrants to the EU/EEA.

## 2. Materials and Methods

The Campbell and Cochrane Collaboration Equity Methods Group and review team including clinicians, public health experts, and researchers from across the EU/EEA used the Grading of Recommendations Assessment, Development and Evaluation (GRADE) approach to conduct evidence reviews. Additional details of the methods can be found in registered systematic review protocol published in BMJ Open [16].

HBV was selected as a key infectious disease by the review team. The review group followed the PRISMA reporting guidelines [17] for the reporting of this systematic review. In summary, the review team developed key research questions (PICO: Population, Intervention, Comparison, Outcome) and a logic model showing an evidence chain to identify key concepts, to consider the potential role of indirect evidence related to populations and interventions and to support the formulation of search strategies [16].

The review teams aimed to answer the following overarching questions:

- Is screening for HBV infection (and subsequent management) associated with decreased morbidity and mortality in migrant populations?
- What is the effectiveness of HBV vaccination programs in migrant populations?
- What is the cost-effectiveness of screening and vaccination programs for HBV?

We used a combination of key terms including “hepatitis B”, “prevalence”, “screening”, “cost”, “efficacy”, and “harms”. See Appendix 1 for the complete list of search terms. Evidence specific to migrants and the EU/EEA was prioritized, but evidence was considered regardless of the population. In particularly whenever possible, we sought to include studies examining marginalized communities, or those with limited health care access, which may be more comparable to migrants than the general population. Migrants included asylum seekers, refugees, undocumented migrants, and other foreign-born residents with a focus on those recently arrived.

We searched MEDLINE via OVID and EMBASE, and NHS EED for evidence on the effectiveness of screening and the cost-effectiveness of screening between 1 January 2010 and 31 December 2016. Finally, we searched the CEA Registry (Tufts University) and Google Scholar databases for additional evidence on cost-effectiveness. For the purpose of this review we considered English language systematic reviews, randomized control trials, and economic studies, evaluating testing, vaccination, and treatment.

Two independent team members (DM & EA) reviewed the titles and abstracts identified by the search. Disagreements were resolved by consensus. The full text of identified citations was then screened for inclusion (DM & EA) with disagreements resolved by consensus. One reviewer (DM) extracted the data from the included study and a second reviewer (EA) verified the data. The methodological quality of included studies was assessed using AMSTAR (Assessing the Methodological Quality of Systematic Reviews) [18].

For evidence around cost-effectiveness, we independently screened and extracted relevant data from the primary studies including economic study design (e.g., micro-costing study, within-trial cost-utility analysis, decision-analytic model); the intervention and comparator, the difference in resource use, costs and cost-effectiveness (e.g., incremental net benefit or incremental cost-effectiveness ratio), and three specific questions for the GRADE Evidence to Decision table: the size of the resource requirements, the certainty of evidence around resource requirements, and whether the cost-effectiveness results favored the intervention or comparison [19]. Finally, we assessed the certainty of economic evidence in each study using the relevant items from the 1997 Drummond checklist [20].

We assessed the certainty of the quantitative evidence using the GRADE approach. We incorporated evidence from a review of qualitative studies relating to hepatitis B and migrants. On 5 June 2018, we did a pre-publication rapid update search for new high-quality evidence pertaining to our PICO questions.

## 3. Results

**Figure 1:**
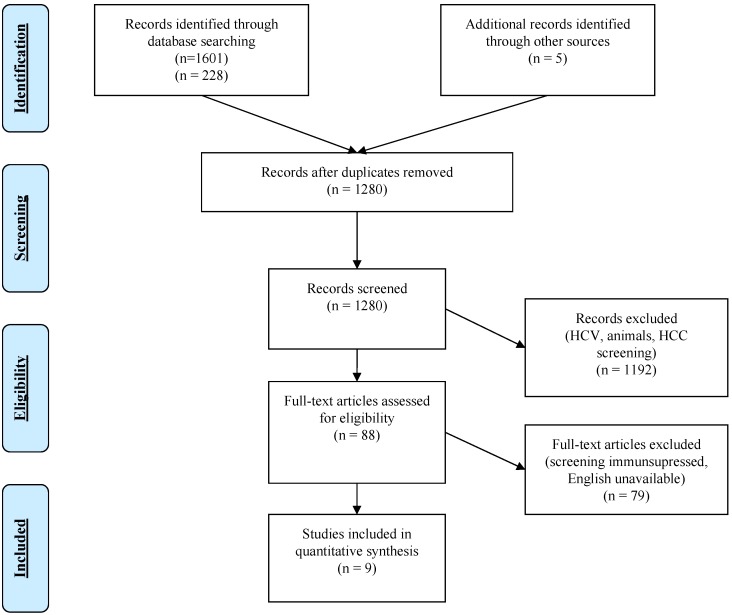
PRISMA Flow Diagram for Effectiveness of HBV screening and vaccination programs * No randomized control studies on screening were identified. Included studies were on topics relevant to the evidence chain (testing, prevalence, vaccination, and treatment).

The search for evidence on effectiveness of screening and vaccination for CHB identified a total of 1829 results. After full text screening, we included four systematic reviews, three guidelines, and two studies, which met our inclusion criteria. See Figure 1 (PRISMA flow chart) and Table 1. List of included studies. Studies were excluded for the following reasons: focus on hepatitis C and not hepatitis B, HCC screening, animal studies and screening for HBV before starting immunosuppressive therapies.

In a rapid update prior to publication, we identified one additional systematic review examining the uptake of screening for HBV in migrant communities.

### 3.1. Effectiveness of Screening for CHB

Screening tests involving serologic markers are considered to have high validity (sensitivity and specificity of greater than 98% for detecting HBsAg) for the detection of CHB infection [2]. Our review found evidence of effective treatment options for CHB that reduced disease morbidity and mortality in specific subsets of patients [22,23]. Treatment guidelines, including whom to treat, when to initiate therapy and the first line agents vary across the EU/EEA [22]. Addressing optimal treatment for CHB was outside the scope of this review. However, the presence of effective treatment options including pegylated IFNα [22] and nucleos(t)ide analogues [21,23] provide indirect evidence that suggests screening is likely to be worthwhile, at least for high-risk populations.

We identified an additional systematic review examining the uptake of screening for CHB among migrants [34]. The review identified four studies examining CHB screening in migrants to the EU/EEA. The median acceptance of HB screening was 87.4% (range 32.3–100%), and 7.3% of migrants were found to have CHB infection (range 0.35–31.8%). The review identified no studies that had examined what percent of migrants identified with CHB were linked to follow up and ongoing care.

### 3.2. Vaccination Against HBV

Despite the inclusion of HB vaccine in many EU/EEA countries National Immunization Programs (NIP), migrant populations may not have access to vaccines due to arrival after the age of vaccination in the general population. Our search and selection did not identify studies examining vaccination programs in migrant populations. However, we did identify evidence from two childhood vaccination programs, one involving marginalized sub populations, within endemic HB communities, demonstrating that childhood vaccination programs were effective at preventing infection. Evidence from a universal infant and childhood vaccination program in Taiwan showed a dramatic decrease in HBsAg seropositivity (9.8–0.7%) between 1984 and 1999 [35,36]. Similarly, a review examining the prevalence of HBV in indigenous and non-indigenous people in the Torres Strait Islands (which is off the coast of mainland Australia) following the implementation of a universal vaccination program for infants and adolescents in 2000 showed a decrease in prevalence of HB infection (6.47% overall prevalence pre 2000 to 2.25%). The impact was particularly pronounced for the indigenous populations who also faced access barriers to health care (16.72% prevalence pre 2000 to 3.96%) [24]. See Table 2 for a summary of evidence for HB vaccination strategies and Box 1 for the GRADE grades of evidence.

Box 1 **GRADE Working Group grades of evidence.**

**High quality:** We are very confident that the true effect lies close to that of the estimate of the effect

**Moderate quality:** We are moderately confident in the effect estimate: The true effect is likely to be close to the estimate of the effect, but there is a possibility that it is substantially different

**Low quality:** Our confidence in the effect estimate is limited: The true effect may be substantially different from the estimate of the effect

**Very low quality:** We have very little confidence in the effect estimate: The true effect is likely to be substantially different from the estimate of effect

Analysis of children vaccinated against HBV in Taiwan demonstrated a decline in the annual incidence of HCC in children 6 to 14 years of age from 0.70 per 100,000 children between 1981 and 1986 to 0.57 between 1986 and 1990, and to 0.36 between 1990 and 1994 (*P* < 0.01). The corresponding rates of mortality from HCC also decreased [25]. We found no studies directly examining the impact of vaccinating susceptible adult migrants against HB.

Surveillance data following vaccination with HBV have not demonstrated significant adverse events [37,38]. In 43,618 Alaskan Natives who received 101,360 doses of HB vaccine, possible adverse reactions occurred in 39 persons and none of the adverse reactions were considered severe [38].

### 3.3. Cost-Effectiveness of Screening and Subsequent Management for Hepatitis B

**Figure 2:**
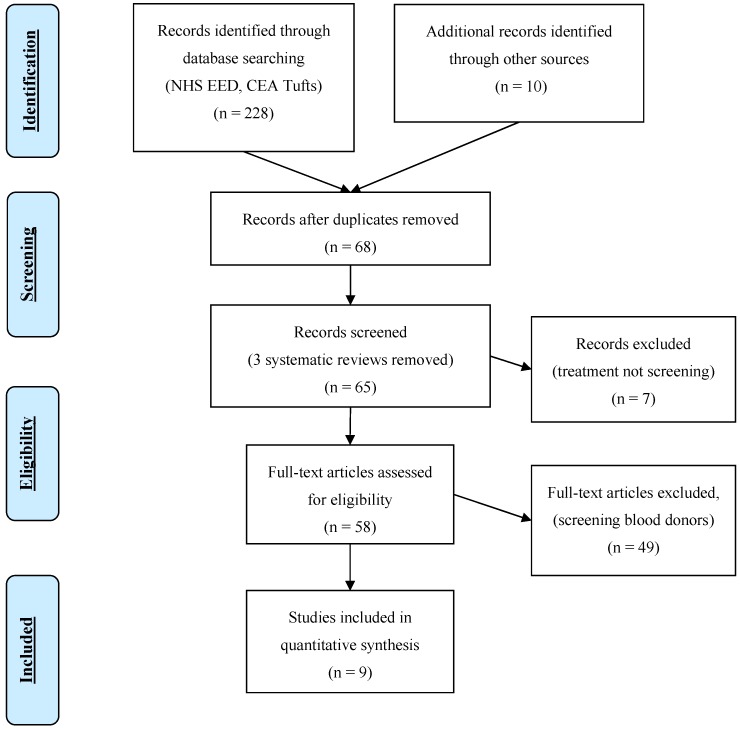
PRISMA Flow Diagram for Cost-Effectiveness of HBV screening.

We retrieved a total of 228 articles from the National Health System Economic Evaluation Database (NHS EED) and the Cost-Effectiveness Analysis Registry at Tufts University (CEA Tufts), and a further ten studies from the effectiveness search from the title and abstract screening. After full text review, we included nine primary studies from our search, see Figure 2 (PRISMA flow chart). Six of the included studies were specific to migrant populations, one of which was conducted in The Netherlands [28], while three were from the USA and two from Canada. Nine primary studies compared the costs and benefits of different strategies including no screening (n = 6); screening and treating (n = 6); screening, treating, and vaccinating (n = 4); universal vaccination (n = 3), and ring vaccination (n = 1).

The review identified one modelling study from the EU/EEA conducted in the Netherlands. The study modelled a cohort of people who either experienced the natural history of HBV infection or received antiviral treatment reported that one-off screening for HBsAg and treating active cases of CHB with Entecavir, resulted in an incremental cost-effectiveness ratio (ICER) of screening and treatment compared with no formal screening, of €8966 per quality-adjusted life year (QALY) gained, with the range of €7222 to €15,694 in sensitivity analysis. These values are below the commonly-used Dutch cost-effectiveness threshold of €20,000 per QALY gained [28].

Among the five studies of migrants to North America, the costs ranged from CAN$6077 [26] to US$86,620 [12] per person screened (and treated in the event of a positive result), with the majority of studies estimating program costs of >$20,000 per person per year. Thus, the costs of these interventions were generally considered moderate. The ICER of screening and treatment for HBV, compared to no screening, ranged from US$36,088 [12] to CAN$40,880 [28] and CAN$101,513 (€72,508) [27] per quality-adjusted life year (QALY) gained. Screening was cost-effective at the host countries’ commonly accepted willingness to pay thresholds. Therefore, all included studies favored screening and treatment for HBV over the status quo of no (or voluntary) screening. Two studies found that HBV screening was likely cost-effective for populations with a prevalence of CHB ≥2% [27]. One study of outpatients to US hospitals found that screening may be cost-effective even in populations with lower than 2% prevalence (i.e., 0.3%) [28].

### 3.4. Cost-Effectiveness of Vaccination

Three studies from North America reported the cost-effectiveness of HBV vaccination, either as a universal strategy or in addition to screening and treatment in mostly migrant populations with the majority arriving from South Asia and Sub-Saharan Africa. The universal vaccination strategy was dominated (i.e., slightly more expensive and slightly less effective) than a no screening intervention in two studies [12,39]. When examining screening for prior immunity and vaccination, all three studies found that strategies adding vaccination of susceptible migrants were not cost-effective or were dominated by the screen and treat strategy [12,26,27]. One of the studies, a US study of Asian and Pacific Islander adult migrants, found that including screening close contacts of infected persons and vaccinating susceptible contacts was cost-effective with an ICER of $39,903 per QALY gained, compared to a screen-and-treat strategy (ICER $36,088/QALY) [12].

## 4. Discussion

Our review found evidence of effective HB vaccination programs for children and adolescents in endemic communities with limited access to care. In addition, we found evidence for effective treatments for cases of CHB that decrease long-term complications in a subset of patients with CHB. A high percentage of migrants to the EU/EEA accept screening for CHB when offered, and this screening successfully identifies cases. Our review found cost-effectiveness studies examining the effect of screening migrants and other populations for HBV infection versus no screening on clinical outcomes.

Our study did not identify any studies examining the impact of vaccination programs on migrant populations. However, we found evidence that vaccination programs are highly effective at reducing disease prevalence and some complications in the general population [35]. In addition, evidence from New Zealand demonstrated that individuals with access barriers to care gain the most from vaccination programs [24]. Vaccination programs targeting migrant children and adolescents and efforts to link migrants to existing national vaccination programs would likely confer similar reductions in disease burden.

More community based and integrated multi-disease screening studies and related cost-effectiveness studies on migrant populations are required to determine the optimal approach to improve uptake and linkage to care. Studies in the EU/EEA on migrant groups with a high prevalence of CHB infection are needed to build trust and knowledge to support the testing approach. Research is needed to ensure vaccination programs reach all migrant children and youth.

The economic literature suggests that screening programs for HBV to identify susceptible individuals or cases and provide treatment are highly likely to be cost-effective in populations with a prevalence of HBV ≥2%, and may be cost-effective at a prevalence as low as 0.3% [27,28]. This finding aligns with studies that support screening for Hepatitis C virus. The evidence for cost-effectiveness of ring vaccination for close contacts is limited, with one study suggesting cost-effectiveness [12].

### 4.1. Implementation Considerations

Qualitative evidence suggests that implementing screening programs and ensuring linkage to treatment and care for migrants presents a number of challenges including: limited access to health care, inability to navigate a complex health care system and cultural and linguistic barriers [40,41]. Migrant populations have identified stigma, lack of access to primary care or testing, and false and confusing information regarding testing and treatment eligibility as significant barriers to accessing screening, vaccination, and treatment [42,43]. For example, a study of predominantly Turkish migrants in the Netherlands found that those who did not speak Dutch were less likely to attend follow up appointments [41]. Nevertheless, the majority of migrants appear to accept screening when offered and studies have suggested they would prefer an integrated screening program to test for multiple diseases simultaneously [43,44,45].

Migrants and other populations from high CHB prevalence countries would potentially benefit from CHB screening in opportunistic facility and community based settings [46]. Facility-based testing occurs when screening is offered to individuals accessing health care for reasons unrelated to HBV. Two examples of offering opportunistic HBV screening for migrants presenting for unrelated reasons to primary health care clinics in Italy found that greater than 90% of migrants accepted screening for HB when offered [47,48]. Community-based screening, in which screening occurs outside health care facilities and community members participate in the programs design and implementation, offers an opportunity to screen patients who may not otherwise present for health care, but this approach is more resource intensive than facility-based testing [46]. A study of Chinese migrants in the Netherlands offered screening in schools, community centers and churches or at the local public health clinic. The study screened 1090 of the estimated 8000 Chinese individuals living in Rotterdam over a 3-month period with the majority preferring to be screened in a community setting [49]. However, in a community based mosque screening program in UK without Pakistani community engagement, no patients presented for screening [50]. Approaches resulting in low uptake raise concerns about missing marginalized populations, such as migrants. Strategies to promote successful linkage of cases to care for migrants with CHB should be a priority for all programs [41,51]. Successful implementation of HBV screening depends on factors relevant to the local health systems and population. Deciding on the best way to implement screening programs may fall to local public health officials and health systems planners.

### 4.2. Strengths and Weaknesses

Our review has several strengths: the use of a systematic review with a GRADE approach allows for evaluation of the certainty and strength of the best available evidence. In addition, the vaccination studies included both CHB prevalent and marginalized populations, such as an indigenous population in New Zealand.

Our review also has several limitations: we found no systematic reviews or RCTs that directly examined the efficacy of screening migrants for HBV. Instead, we followed an evidence chain approach to estimate the effectiveness of testing and the effectiveness of treatment. The evidence for linkage to HBV care and treatment and existing screening programs for migrants in the EU/EEA was also very limited. There are evidence gaps within the screening and vaccination programs. While testing and treatment for CHB were promising, there is an undeniable evidence gap between linkage to care and treatment for migrants. The vaccination programs that we identified targeted various high-risk and sometimes marginalized children. Studies on high-risk migrant populations were limited.

The number of cost-effectiveness studies identified was limited and mostly related to migrants in North America. Economic evidence is most relevant to the health system in which the study was undertaken, and therefore, certain economic evidence may not be transferable to EU/EEA. It was not possible to accurately convert from US or Canadian dollars to Euros, as the reference year for costs was not adequately reported in all primary studies. In addition, costs may change over time. Not all economic studies assessed the levels of seroprevalence across plausible ranges in their sensitivity analyses, limiting the generalizability of the cost-effectiveness results. Furthermore, most studies used static decision tree models, which assume a constant probability of acquiring an HBV infection, limiting the model’s ability to accurately predict cost-effectiveness. Test sensitivity and specificity was not clearly described in some studies. Definitive economic analysis was limited as most included studies only considered screening for HBV in isolation rather than an integrated multi-disease screening program for HBV, HCV, and HIV.

## 5. Conclusions

Migrants arriving or living in the EU/EEA who originate from HBV endemic countries, have an increased burden of CHB compared to the general population. Screening high-risk migrants for HBV and offering monitoring and treatment to those found to be chronically infected will offer clinical benefits. HBV vaccination programs targeting marginalized and high-risk children and adolescents significantly reduces the prevalence of CHB. A strategy of screening and treating CHB compared to no screening is likely to be cost-effective. Cost-effectiveness of screening increases with increasing HB seroprevalence and uptake, but programs may be cost-effective even in lower seroprevalence groups (<2%). Qualitative evidence suggests that developing screening approaches for migrants will be challenging as migrants often lack access to primary health care and may face additional barriers to care. A mixture of vaccination and testing programs, in a variety of settings, with an emphasis on the linkage of positive cases to care will most likely have the greatest impact.

## Figures and Tables

**Table 1(a):** Characteristics of included studies for screening and vaccine program effectiveness for Hepatitis B Virus.

Study	Quality	Type of Study	Population	Intervention	Results/Outcomes
**Should Hepatitis B Virus (HBV) screening be offered to recently arrived migrants to the EU/EEA?**					
Chou et al. 2014 [21]	AMSTAR * 9/11	Systematic Review	12 RCTs	Treatment with Nucelos(t)ide Analogues (NAs) compared to placebo	Reduced rate of intermediate outcomes in NA group (HBV DNA loss, HBeAg loss, Histologic improvement, HBsAg loss). No significant decrease in HCC incidence. No increase in significant adverse events in NA group but higher rates of study withdrawal
Wong et al. 2010 [22]	AMSTAR 7/11	Systematic Review	11 RCTs	Treatment with pegylated interferon alpha compared to placebo	Decreased rate of Hepatic Events (RR 0.55 95% CI 0.43 to 0.70), cirrhotic complications (RR 0.46 95% CI 0.32 to 0.67) and liver related mortality (RR 0.63 95% CI 0.42 to 0.96) for treatment group
EASL 2017 [23]	NA	Guideline			Current Treatment Guidelines for acute and chronic infection with hepatitis B for the EU/EEA
ECDC 2016 [3]	NA	Technical Document	EU/EEA Migrants/General Population	No intervention	5.5% of migrants born in intermediate/high prevalence countries infected with CHB infection compared to 1.12% in the general population of EU/EEA Migrants from HBV endemic countries 5.1% of population of the EU/EEA but 25% (range 14–47%) of the total number of CHB cases
WHO 2017 [2]	NA	Guideline			Guidelines on hepatitis B testing including information on implementation of screening programs.
WHO 2015 [1]	NA	Guideline			Guidelines for the prevention, care and treatment of persons with chronic hepatitis B infection.
What is the effectiveness of vaccination programs against HBV?					
Graham et al. 2013 [24]	AMSTAR 4/11	Systematic Review	Australia: Aboriginal population/general population	Vaccination with HBV vaccine compared to no vaccine	Reduced rate of positive HBsAg in post vaccination cohort 3.96% (95% CI: 3.15–4.77) compared to pre-vaccination cohort 16.72 (95% CI: 7.38–26.06).
Chang et al. 2009 [25]	NA	Individual Study	Endemic Country Taiwan (Republic of China)	Vaccination with HBV vaccine compared to no vaccine	Reduced rate of positive HBsAg (RR 0.07 95% CI 0.04 to 0.13) and chronic liver disease mortality (RR 0.34 95% CI 0.25 to 0.45) in post vaccination cohort
Rossi et al. 2012 [8]	AMSTAR 6/11	Systematic Review	Global Migrant Population	No intervention	Prevalence of CHB higher in refugees and asylum seekers compared to immigrants (9.6% vs. 5.1%). 39.7% of migrants demonstrated prior immunity to HBV either through prior infection or vaccination (95% CI: 35.7–43.9%).

* A Measurement Tool to Assess Systematic Reviews (AMSTAR).

**Table 1(b):** Characteristics of included studies cost-effectiveness of screening for HBV.

Study	Certainty of Economic Evidence (Quality)	Design	Population	Intervention	Cost-Effectiveness	Resource Requirements
What is the cost-effectiveness of screening (and subsequent management) migrants for chronic hepatitis B (CHB)?						
Rossi et al. 2013 [26]	Allowance was made for uncertainty in the estimates of costs and consequences across plausible ranges. Appropriate statistical analyses (probabilistic sensitivity analyses (PSA) were performed for costs and consequences. Justification was provided for key study parameters and some upper and lower range estimates. Some ranges cited the data sources; other ranges provided an assumption for upper and lower limits but no further justification. The cost-effectiveness results were not very sensitive to changes in the values. Most of time, the intervention was still cost-effective. At a standard willingness to pay threshold, the probability of being cost-effective was 78% (high).	Decision-analytic Markov model, results presented in Canadian dollars.	Vaccination strategies for newly arrived adult Canadian immigrants and refugees	(i) universal vaccination, (ii) screening + vaccination (iii) screening+ treatment (iv) screening +treatment + vaccination	(i) and (ii) were dominated by no intervention (iii) screening and treatment: CAN$40,880 per QALY gained vs no intervention (iv) screen, treatment, vaccination: CAN$437,335 per QALY gained vs no intervention	The intervention has moderate costs. Categories and volumes of resource use were not reported separately.
Wong et al. 2011 [27]	All the ranges were provided. Both one way, and probabilistic sensitivity analyses were performed. All data sources for model inputs were provided, with the exception of reference years for all costs. The results were sensitive to the progression rate and discount rate used. The intervention had a 55% probability of being cost-effective compared to a no screening strategy. Certainty in the results was deemed to be moderate overall.	Decision-analytic Markov model; reported in Canadian dollars	Immigrants to Canada	(i) ‘No screening’; (ii) ‘Screen and Treat’ (iii) ‘Screen, Treat and Vaccinate’	ICER for Screen and treat ranged from CAN$45,221 (Tenofovir, 3% discount) per QALY gained to CAN$101,513 (Entecavir, 5% discount) ICER for Screen, treat and vaccinate ranged from CAN$96,523 (Entecavir, 3% discount) to CAN$3,648,123 per QALY gained (Tenofovir, 5% discount) Favours intervention (ii): screen and treat, CAN$69,209/QALY gained (cost-effective) A vaccination program following the screening program was not cost-effective compared with the screen and treat strategy.	Large costs for strategy ii: screen and treat if using either Entecavir or Tenofovir. Large costs for strategy iii: screen, treat and vaccinate using Entecavir. Other interventions had moderate costs. Resource use was not quantified separately.
Hutton et al. 2007 [12]	Allowance for uncertainty was accommodated. Both one way and PSA were conducted. All the data sources were provided for ranges used in sensitivity analyses. The probability of being cost-effective was 82–85% when the values of key variables were changed. Therefore the certainty of the intervention being cost-effective was deemed high.	Decision-analytic Markov model; results in US dollars	Asian and Pacific Islander adult immigrants to the US	(i) No Screening: universal vaccination strategy for all individuals (ii) Screen, Treat and No Vaccination: a screen-and-treat strategy: screen individuals and treat infected persons; (iii) Screen, Treat and Vaccinate: vaccine for non-infected persons (iv) Screen, Treat and Ring Vaccinate: screen for close contacts and vaccinate non-infected persons	The screen-and-treat strategy, intervention (ii) has an incremental cost-effectiveness ratio of US$36,088 per QALY gained compared with the status quo. Screen and treat and ring vaccinate strategy, intervention (iv) has a cost-effectiveness ratio of US$39,903 per QALY gained compared with the screen-and-treat strategy. Universal vaccination, intervention (i) and screen and treat and vaccine were dominated.	Costs were moderate, ranging from US$85,000 per person per lifetime with universal vaccination to US$87,000 per person per lifetime to screen, treat and ring vaccinate.
Veldhuijzen et al. 2010 [28]	Uncertainty was tested and all ranges were provided Univariate, multivariate, PSA were conducted. Data sources for all the ranges were provided Cost-effectiveness results were robust to changes in model parameters. The probability of being cost-effective was 72%; the certainty was deemed moderate.	Decision-analytic Markov model; results reported in Euros	Migrants to the Netherlands from intermediate and high HBV endemic areas	One-off systematic screening and subsequent treatment of eligible patients, compared with the status quo: i.e., existing pregnancy screening, testing due to medical complaints, contact tracing, and checkup for STIs.	Incremental cost-effectiveness ratio (ICER) of €8966 per QALY gained. Discounted costs at 4% and effects at 1.5%, resulted in a slightly lower ICER of €8823 per QALY gained.	Status quo had low test costs at €458 while the screen and treat strategy had a test cost of €15,954. Referral and follow up costs for the status quo strategy was €838; while the screen and treat strategy had a follow up cost of €3074. i.e., a large difference. Screening and treatment costs per person were ~€130 per person.
Rein et al. 2011 [29]	Standard deviations were provided for costs.	Costing study (exploratory, pilot study)	Overseas-born community living in the US	Screening models: (i) Community Clinic (ii) Community Outreach (iii) Outreach partnership (iv) Partnership contract	Cost-effectiveness was not reported	Cost per complete screen ranged from US$40 for the Community Clinic to US$280 for the Partnership Contract model. Low costs for Community based but higher costs for Partnership model. The costs per positive person identified varied from US$609 in the Community model to US$4657 in the Partnership model. Cost per complete screen/cost per newly identified positive case (adj. for prevalence): 1) US$40/$854 ($895) 2) US$102/$2641 ($2698) 3) US$280/$6300 ($6013) 4) US$176/$5709 ($5063)
Jazwa et al. 2015 [30]	Allowance was made for uncertainty, all the ranges were provided. Not all statistical tests were reported. Univariate sensitivity analysis was conducted, which is consistent with the study design (cost benefit study, not cost-effectiveness). All the data sources and assumptions were provided. Not applicable.	Cost-benefit analysis	Refugees to the US; costs reported in US dollars	(i) Vaccinate only without HBV screening (ii) Screen, then vaccinate or initiate management	The net benefits of the screen and vaccinate strategy ranged from US$24 million to US$130 million after 5 years from program initiation	The cost per refugee for the vaccination only strategy was low if the screen rate <70%, however after 10 years, if the screening rate was more than 70%, the cost of the vaccination only strategy was moderate: US$706–$968 per person.
Ruggeri et al. 2011 [31]	Allowance was made for uncertainty, all the ranges were provided. Both one way and PSA were conducted. All the data sources and assumptions were provided. The results were not sensitive to changes in the model values. The probability of being cost-effective was 70–98%; the certainty of the results was deemed high.	Decision-analytic Markov model; results reported in Euros	Residents of Italy	(i) Screening of Italian patients at risk (assumed prevalence of 7%) and treatment of cases according to protocol; (ii) compared with no screening and treatment of patients with cirrhosis or HCC	ICER of €18,256 per QALY gained (±€387) for screening compared to no screening	High costs for the screening strategy: €67,008 (±€515) per person per year; Low cost for no screening strategy, but moderate costs for the screen and treat strategy. Moderate cost for no screening strategy: €7939 (±€1679) per person per year
Eckman et al. 2011 [32]	Allowance was made for uncertainty, all the ranges were provided. PSA was conducted All the data sources and assumptions were provided. The cost-effectiveness results were sensitive to model parameters including cost of treatment, drug resistance, and disease prevalence. The probability of the intervention being CE was 49%; certainty was deemed moderate.	Decision-analytic Markov model; results reported in US dollars	Asymptomatic outpatients in the US	Screening for Hepatitis B surface antigen followed by treatment of appropriate patients with (i) pegylated interferon-a2a for 48 weeks, (ii) a low-cost nucleoside or nucleotide agent with a high rate of developing viral resistance for 48 weeks, (iii) prolonged treatment with low-cost, high-resistance nucleoside or nucleotide, (iv) prolonged treatment with a high-cost nucleoside or nucleotide with a low rate of developing viral resistance; compared with no screening	Intervention (iii) was dominated by the no screening intervention; Intervention (ii) and intervention (v) were dominated by intervention (iv). Intervention (iv) was cost-effective with an ICER of US$29,232 per QALY gained.	Low cost for no screening strategy US$915 per person per year. Moderate cost for screen and treat, ranging from US$1170 (treat with low cost, high resistance nucleoside) to US$1286 (treat with high cost, low resistance nucleoside). Resource use was not reported separately.
Li et al. 2013 [33]	Allowance was made for uncertainty. All upper bound and lower bound limits were provided. Only univariate sensitivity analysis was conducted. No, justification was provided for the ranges tested for price of treatment, or probability of disease progression. The cost-effectiveness results were not sensitive (i.e., remained robust) to changes in the values of variables. Moderate certainty.	Decision-analytic Markov model; results reported in US dollars.	Residents of Zhoushan Island in mainland China.	Monitor and treat scenarios in 3 patient groups according to treatment eligibility, (1) ineligible (2) borderline (3) eligible compared with natural history (no screening and no antiviral treatment of patients with cirrhosis or HCC)	ICER of the monitor and treat strategy compared to the natural history was US$97 per QALY gained for the ineligible group, US$500/QALY for the borderline group, US$1131/QALY for the eligible group. With a 5% reduction in Entecavir price: the monitor and treat strategy becomes cost saving (ICER < 0) in the ineligible group; the ICER was US$254 for the eligible group, and US$860 for the eligible group. With a 50% reduction in Entecavir price: the monitor and treat strategy was cost saving for all sub groups: (ICER < 0).	For the ineligible group: Difference in costs was small. For example, total costs per patient per lifetime was US$21,229 for natural history strategy, and US$21,550 for Monitor and Treat. For the borderline group: The difference in costs was larger. For natural history strategy, the total costs = per patient lifetime was US$33,280 while the total cost per patient lifetime for the monitor and treat strategy was US$37,043. For the eligible group: The difference in costs was largest. With natural history strategy, total cost per patient lifetime was US$32,430 while total cost per patient lifetime for monitor and treat strategy was US$42,711.

**Table 2:** GRADE Evidence Profile: Effect of HBV vaccination on preventing HBV infection, chronic liver disease and HCC.

Certainty Assessment							Summary of Findings				
№ of participants (studies) Follow-up	Risk of bias	Inconsistency	Indirectness	Imprecision	Publication bias	Overall certainty of evidence	Study event rates (%)		Relative effect (95% CI)	Anticipated absolute effects	
							With no vaccine	With HBV vaccine		Risk with no vaccine	Risk difference with HBV vaccine
**HCC mortality**											
54289638 (1 observational study)	serious ^a^	not serious ^b^	not serious	serious ^c^	none	Very Low	135/27144819 (0.0%)	20/27144819 (0.0%)	RR 0.90 (0.75 to 1.09)	0 per 100,000	0 fewer per 100,000 (0 fewer to 0 fewer)
**Liver cancers (except non-hepatocellular carcinoma)**											
6898803 (1 observational study)	serious ^a^	serious ^b^	not serious	serious ^c^	none	Very Low	24/3381519 (0.0%)	20/3517284 (0.0%)	RR 0.80 (0.42 to 1.48)	1 per 100,000	0 fewer per 100,000 (0 fewer to 0 fewer)
**HBsAg carriage**											
1916 (1 observational study)	serious ^d^	not serious	not serious	not serious	strong association ^e^	Low	39/559 (7.0%)	9/1357 (0.7%)	RR 0.07 (0.04 to 0.13)	6977 per 100,000	6488 fewer per 100,000 (6698 fewer to 6070 fewer)
**Anti-HBc**											
1916 (1 observational study)	serious ^d^	not serious	not serious	not serious	strong association ^e^	Low	115/559 (20.6%)	39/1357 (2.9%)	RR 0.11 (0.08 to 0.16)	20,572 per 100,000	18,309 fewer per 100,000 (18,927 fewer to 17,281 fewer)
**Chronic Liver Disease**											
54289638 (1 observational study)	serious ^a^	not serious	not serious	not serious	strong association	Low	407/38702888 (0.0%)	55/15586750 (0.0%)	RR 0.34 (0.25 to 0.45)	1 per 100,000	1 fewer per 100,000 (1 fewer to 1 fewer)
**HCC Incidence**											
54289638 (1 observational study)	serious ^a^	not serious	not serious	not serious	none	Very Low	712/38702888 (0.0%)	191/15586750 (0.0%)	RR 0.89 (0.75 to 1.04)	2 per 100,000	0 fewer per 100,000 (0 fewer to 0 fewer)
**HBsAg (continuous)**											
8545 (8 observational studies)	serious ^a^	serious ^f^	not serious	not serious	none	Very Low	5516 (Number of Events)	3029 (Number of Events)	N/A	The mean HBsAg (continuous) ranged from 5.19–25.99 %	3.96 % lower (3.15 lower to 4.17 lower)

^a^ Cohort Study, downgraded due to risk of bias; ^b^ Heterogeneity is not reported; ^c^ Large confidence intervals; ^d^ Cohort Study, risk of bias was not assessed; ^e^ Large effect; ^f^ Hetegeniety (I-squared: 94.9%); **CI:** Confidence interval; **RR:** Risk ratio. **Bibliography:** Ni YH, Chang MH, Huang LM, et al. Hepatitis B virus infection in children and adolescents in a hyperendemic area: 15 years after mass hepatitis B vaccination. Ann Intern Med 2001;135:796-800 Chang M-H, Chen C-J, Lai M-S, et al. Universal hepatitis B vaccination in Taiwan and the incidence of hepatocellular carcinoma in children. NEngl J Med 1997;336:1855-9. Graham S, Guy RJ, Cowie B, Wand HC, Donovan B, Akre SP, et al. Chronic hepatitis B prevalence among Aboriginal and Torres Strait Islander Australians since universal vaccination: a systematic review and meta-analysis. BMC Infect Dis. 2013 Dec;13(1):403. Chang MH, You SL, Chen CJ, Liu CJ, Lee CM, Lin SM, et al. Decreased incidence of hepatocellular carcinoma in hepatitis B vaccines: A 20-year follow-up study. J Natl Cancer Inst. 2009;101(19):1348–55.

## Appendix 1. Search Terms

Database: Ovid MEDLINE(R) Epub Ahead of Print <May Week 3 2016>, Ovid MEDLINE(R) 1946 to Present with Daily Update.

Search Date: 26 May 2016

--------------------------------------------------------------------------------

1 exp Hepatitis B/(49954)
2 (CHB or HBV or HepB).mp. (32246)
3 ((hep or hepatitis) adj3 B).mp. (82681)
4 hbsag.tw. (16191)
5 (hbs adj2 ag).tw. (720)
6 hb-s-ag.tw. (24)
7 ((serum or type b) adj2 hepatitis).tw. (3299)
8 or/1–7 (86774)
9 exp Mass Screening/(108496)
10 (screened or screening? or tested or testing or tests).tw. (1733475)
11 Early Diagnosis/(19328)
12 ((case? or early) adj2 (detected or detection? or diagnos$ or discover$)).tw. (153478)
13 exp Population Surveillance/(56663)
14 (disease? adj2 surveillance).tw. (4191)
15 Contact Tracing/ (3561)
16 contact tracing.tw. (1176)
17 or/9–16 (1940435)
18 meta analysis.mp,pt. (96656)
19 review.pt. (2060002)
20 search$.tw. (266555)
21 guideline.pt. (15761)
22 guideline/(15761)
23 guidelines as topic/(34049)
24 practice guideline.pt. (21200)
25 practice guideline/(21200)
26 practice guidelines as topic/(91709)
27 (CPG or CPGs or guidance or guideline? or recommend$ or standard?).ti. (147079)
28 exp clinical pathway/(5268)
29 exp clinical protocol/(139279)
30 ((care or clinical) adj2 pathway?).tw. (5122)
31 or/18-30 (2570832)
32 8 and 17 and 31 (2192)
33 animals/ not (humans/ and animals/) (4214239)
34 32 not 33 (2176)
35 34 and (2010$ or 2011$ or 2012$ or 2013$ or 2014$ or 2015$ or 2016$).ed. (675)
36 remove duplicates from 35 (reviews and guidelines) (652)
37 exp “costs and cost analysis”/ (197842)
38 cost$.mp. (467557)
39 cost effective$.tw. (83015)
40 cost benefit analys$.mp. (67281)
41 health care costs.mp. (37134)
42 or/37–41 (476890)
43 8 and 17 and 42 (888)
44 animals/not (humans/and animals/)(4214239)
45 43 not 44 (883)
46 45 and (2010$ or 2011$ or 2012$ or 2013$ or 2014$ or 2015$ or 2016$).ed. (266)
47 remove duplicates from 46 (costing) (254)

Database: Embase <1974 to 2016 May 26>

Search Date: 26 May 2016

--------------------------------------------------------------------------------

1 exp hepatitis B/(79653)
2 (CHB or HBV or HepB).mp. (53611)
3 ((hep or hepatitis) adj3 B).mp. (132717)
4 hbsag.tw. (24116)
5 (hbs adj2 ag). tw. (1126)
6 hb-s-ag.tw (626)
7 ((serum or type b) adj2 hepatitis).tw. (4201)
8 or/1–7 (140818)
9 exp mass screening/(182894)
10 (screened or screening? or tested or testing or tests).tw. (2429779)
11 anonymous testing/(223)
12 early diagnosis/(83109)
13 ((case? or early) adj2 (detected or detection? or diagnos$ or discover$)).tw. (235744)
14 exp health survey/(184232)
15 (disease? adj2 surveillance).tw. (5252)
16 contact examination/(2867)
17 contact tracing.tw. (1512)
18 or/9-17 (2853474)
19 meta analysis.mp,pt. (163362)
20 review.pt. (2163167)
21 search$.tw. (371891)
22 guideline.pt. (0)
23 guideline/(144)
24 guidelines as topic/(229891)
25 practice guideline.pt. (0)
26 practice guideline/(275498)
27 practice guidelines as topic/(171087)
28 (CPG or CPGs or guidance or guideline? or recommend$ or standard?).ti. (203281)
29 exp clinical pathway/(6983)
30 exp clinical protocol/(75932)
31 ((care or clinical) adj2 pathway?).tw. (9455)
32 or/19-31 (2897811)
33 8 and 18 and 32 (3886)
34 (exp animal/ or animal.hw. or nonhuman/) not (exp human/ or human cell/ or (human or humans).ti.) (5865316)
35 33 not 34 (3822)
36 (immigra$ or migrant$ or migration$ or refugee$).mp. (337937)
37 35 and 36 and (2010$ or 2011$ or 2012$ or 2013$ or 2014$ or 2015$ or 2016$).dd. (68)
38 remove duplicates from 37 (reviews and guidelines) (67)
39 cost effectiveness analysis/(114261)
40 cost.tw. (387424)
41 costs.tw. (208729)
42 or/39-41 (544762)
43 8 and 18 and 42 (1580)
44 (exp animal/ or animal.hw. or nonhuman/) not (exp human/ or human cell/ or (human or humans).ti.) (5865316)
45 43 not 44 (1556)
46 (immigra$ or migrant$ or migration$ or refugee$).mp. (337937)
47 45 and 46 and (2010$ or 2011$ or 2012$ or 2013$ or 2014$ or 2015$ or 2016$).dd. (59)
